# Cytokine profiling identifies circulating IL-2, IL23 and sPD-L1 as prognostic biomarkers for treatment outcomes in non-small cell lung cancer patients undergoing anti-PD1 therapy

**DOI:** 10.3389/fonc.2025.1628379

**Published:** 2025-07-08

**Authors:** Kriti Jain, Anika Goel, Deepa Mehra, Deepak Kumar Rathore, Akshay Binayke, Shyam Aggarwal, Surajit Ganguly, Amit Awasthi, Evanka Madan, Nirmal Kumar Ganguly

**Affiliations:** ^1^ Department of Biotechnology and Research, Sir Ganga Ram Hospital, New Delhi, India; ^2^ Immunology Lab, Translational Health Science and Technology Institute, Faridabad, India; ^3^ Medical Oncology, Sir Ganga Hospital, New Delhi, India; ^4^ Department of Molecular Medicine, Jamia Hamdard, New Delhi, India

**Keywords:** non-small cell lung cancer (NSCLC), immune checkpoint inhibitors (ICIs), IL-2, sPD-L1, IL-23, biomarkers, nivolumab

## Abstract

**Background:**

This study investigates the predictive potential of circulating cytokines for response and survival outcomes in patients with advanced non-small cell lung cancer (NSCLC) undergoing immune checkpoint inhibitor (ICI) therapy.

**Materials and methods:**

A cohort of 64 patients with advanced NSCLC receiving ICI therapy were included. Baseline serum samples were collected prior to ICI initiation and profiled using a multiplex cytokine panel. Logistic regression, Cox regression, and Kaplan-Meier survival analysis were employed to assess associations between cytokine levels, therapeutic response, progression-free survival (PFS), and overall survival (OS). Gene expression levels of key cytokines were validated in peripheral blood mononuclear cells (PBMCs) of 17 patients (Responders = 7, Non-Responders = 10) and 3 Healthy Controls using quantitative real-time PCR.

**Results:**

Elevated baseline levels of IL-2, IL-23, and sPD-L1 were significantly associated with clinical response to ICI therapy. Among these, sPD-L1 emerged as an independent predictor of response (AUC = 0.87). Multivariate Cox regression showed IL-2 (HR = 0.67), sPD-L1 (HR = 0.15), and IL-23 (HR = 1.18) were significantly associated with PFS and also predictive of OS. Notably, combined profiling of IL-2 and sPD-L1 enhanced predictive power (AUC = 0.95 for both PFS and OS). RT-PCR analysis of PBMCs corroborated these findings, confirming upregulation of IL-2 in responders and elevated IL-23 expression in non-responders.

**Conclusion:**

Baseline cytokine profiling particularly of IL-2, sPD-L1, and IL-23 provides important prognostic and predictive information in advanced NSCLC patients undergoing ICI therapy. These biomarkers may facilitate more personalized approaches to immunotherapy and guide clinical decision-making.

## Introduction

Lung cancer remains one of the leading causes of cancer-related mortality worldwide, affecting both smokers and non-smokers. In India, it is currently ranked as the fourth most common cause of cancer-related deaths, highlighting its aggressive nature and the urgent need for improved therapeutic strategies (1).

Over the past few years, significant advancements have been made in the treatment of lung cancer, evolving from conventional chemotherapy and radiotherapy to more targeted therapies designed to target specific molecular drivers of tumor progression. While these advancements have improved disease management to some extent, their overall effectiveness remains limited, often accompanied by severe side effects and minimal impact on long-term survival (2).

In recent years, immune checkpoint inhibitors (ICIs) therapy has emerged as a groundbreaking approach in lung cancer treatment. These therapies function by stimulating the host immune response, targeting inhibitory pathways such as programmed death-ligand 1 (PD-L1), programmed death-1 (PD-1), and cytotoxic T-lymphocyte-associated protein 4 (CTLA-4), which are exploited by tumor cells to evade immune surveillance. Despite their promising potential, the clinical benefits of ICIs are observed in only 15–45% of patients, highlighting the need for robust predictive biomarkers to guide patient selection (3).

Current biomarkers in clinical use such as PD-L1 expression (3), mismatch repair deficiency (MMR) (4), and tumor mutational burden (TMB) (5) are being used, but they have limitations in accurately forecasting treatment response. This limitation has prompted growing interest in the role of cytokines as potential biomarkers and immunomodulatory agents in lung cancer (6).

Cytokines are essential molecular messengers that regulate immune system communication, enabling a coordinated response against target antigens. While immune signaling often occurs through direct cell-to-cell interaction, cytokines allow for rapid and efficient immune modulation. Their role in cancer treatment has gained significant attention, as they enhance immune cell activation, tumor recognition, and anti-tumor responses. However, due to their pleiotropic functions, redundancy, and capacity for both immune stimulation and suppression, their role in cancer immunity is complex and context-dependent. Understanding this dual nature is essential to harnessing cytokines as therapeutic tools and predictive markers (7).

Investigating cytokine profiles in the tumor microenvironment may offer valuable insights into patient responsiveness to immunotherapy. Their ability to reflect the dynamic interactions between tumor cells and immune components positions them as promising biomarkers for predicting treatment outcomes and guiding clinical decisions in advanced non-small cell lung cancer (NSCLC) (8).

This study aims to explore the potential of cytokines as immunological biomarkers in lung cancer, with a focus on their role in modulating immune responses and predicting patient benefit from immune checkpoint blockade.

## Materials and methods

### Patients and treatment

This study included a total of 64 patients with histologically confirmed metastatic solid tumors in non-small cell lung cancer (NSCLC).All patients were above 18 years of age and were recruited from Sir Ganga Ram Hospital (SGRH), New Delhi between 2019 and 2021. Patients received immune checkpoint inhibitor therapy with Nivolumab. The study was conducted following approval from the Institutional Ethics Committee at Sir Ganga Ram Hospital (Reference No. EC/04/19/1499). Detailed Inclusion and exclusion criteria to enroll patients is as mentioned in **Supplementary Table S1**. In addition to the patient cohort, 30 healthy individuals who volunteered to be a part of the study were also enrolled as the control group, serving as age- and sex-matched healthy controls. The cytokine levels were normalized by the median plasma cytokine levels of healthy volunteers. All patients received standard-of-care immune checkpoint inhibitors as follows: Nivolumab: 200 mg intravenously, administered biweekly.

Peripheral blood samples (~10 mL) were collected from all patients prior to the initiation of ICI therapy (Baseline) using both plain yellow-top tubes and EDTA tubes. These samples were used for detailed immunophenotyping and multi-omics analyses.

### Response assessment

All patients recruited in this study were followed until either disease progression or death. Treatment response was assessed both clinically and radiologically. Radiological evaluation of disease progression was measured using magnetic resonance imaging (MRI) and therapeutic response was evaluated according to the Response Evaluation criteria in Solid Tumors 1.1 (RECIST 1.1). Clinical assessment of response to Nivolumab was performed at 8–12 week intervals. Patients were categorized as responders if they demonstrated complete/partial response with complete or atleast 30% decrease in target lesions, or stable disease wherein the target lesion is not progressing. Non-responders were defined as those who exhibited at least a 20% increase in the sum of diameters of target lesions, and thereby stated as progressive disease.

### Sample collection

#### Blood sample processing- isolation of serum/plasma/PBMCs

##### Isolation of serum

Blood samples were collected in serum separator tubes (SST) or plain yellow-top vials (BD Vacutainer SST tubes, 367989) and allowed to clot at room temperature for 30 minutes. Following clot formation, samples were centrifuged at 1200 rpm for 10 minutes. The resulting serum was carefully isolated and immediately stored at −80 °C until further use.

##### Isolation of peripheral blood mononuclear cells

For immune monitoring, 8 mL of heparinized blood was collected. The samples were diluted 1:1 with Dulbecco’s Phosphate-Buffered Saline (DPBS) (Sigma Aldrich, D8327) and layered over an equal volume of Histopaque-1077 (Sigma Aldrich, 10771). PBMCs were isolated using a density gradient centrifugation technique at 1200 × g for 35 minutes at room temperature. After centrifugation, red blood cells, granulocytes, and platelets settled at the bottom, while PBMCs were present at the plasma–Histopaque interface. The PBMC layer was carefully aspirated without disturbing the other layers. The collected cells were washed with an equal volume of 1× DPBS and centrifuged at 2000 × g for 10 minutes. A second wash was performed with DPBS, followed by centrifugation at 2000 × g for 7 minutes.

The final PBMC pellet was resuspended in freshly prepared freezing medium consisting of dimethyl sulfoxide (DMSO) (Sigma Aldrich, D8418) and fetal bovine serum (FBS) (Corning, 35-015-CV). Cells were cryopreserved and stored in liquid nitrogen until further use.

### Multiplex cytokine profiling

Stored serum samples isolated from whole blood as per the described protocol, were thawed at room temperature prior to analysis. A panel of 15 cytokines- IL-1α, IL-1β, IL-2, IL-4, IL-10, IL-13, IL-15, IL-17, IL-18, IL-21, IL-23, GM-CSF, TNFα, IFN- γ and sPD-L1 was quantified using the Luminex Discovery Kit (Human premixed multi-analyte kit), R& D Systems, LXSAHM-15 on Luminex Platform (Flex Map 3D, ThermoFisher Scientific, USA).

At first, serum samples were diluted 1:10 using the kit-provided diluent and transferred to polypropylene tubes (ELKAY, 2053-001). Each 96-well plate included a 7-point serially diluted standard curve in duplicate, along with 64 patient samples, 8 of which were run in duplicate. Three batches of Luminex panels were required to analyze 200 patient samples and healthy controls. All procedures were conducted according to the manufacturer’s instructions.

Crystals were completely dissolved by warming the reagents to room temperature. The microparticle cocktail containing all 15 cytokine analytes was prepared by centrifuging at 1,000 × g for 30 seconds, vortexing, and then diluting in the supplied diluent buffer.

Briefly, the assay was performed by adding the microparticle cocktail, diluted serum samples, and cytokine standards to the wells of a 96-well plate, followed by incubation for 2 hours at room temperature on a microplate shaker set to 800 rpm. After incubation, the plates were washed and a biotin-conjugated antibody cocktail was added and incubated for 1 hour. Streptavidin-phycoerythrin (PE) was subsequently added, followed by a 30-minute incubation. A final wash was performed, and microparticles were resuspended in wash buffer.

Washing steps were carried out three times using a magnetic microplate washer. Plates were placed on a magnetic base for 1 minute before aspirating the supernatant. After the final wash, 100μL of wash buffer was added to each well, followed by a 2-minute incubation on the shaker (800 ± 50 rpm).

Plates were read within 90 minutes on the Luminex FlexMap 3D analyzer instrument and data were analyzed using xPONENT software. Values falling below the lower limit of quantification were assigned a value of 1/3 of the lower limit of the standard curve (9).

### RNA isolation and real time PCR

Total RNA was isolated from PBMCs of NSCLC patients (at baseline, before the initiation of ICI) and Healthy Controls using the TRIZOL reagent (Invitrogen, Grand Island, NY) and quantified using a Nanodrop ND-1000 spectrophotometer (Thermo Fischer, USA). cDNA was prepared from one micrograms of RNase-free DNase treated total RNA using first-strand cDNA Synthesis Kit (applied biosystems Thermo), as per manufacturer’s instructions, using random hexamer primers. PCR reactions were carried in Applied Biosystems, Real-Time PCR System (Agilent Technologies Stratagene Mx3005P) using PowerUp SYBR Green PCR Master Mix (Thermo Fisher Scientific, USA). The detail of the primers (sequences and annealing temperatures) used is given in **Supplementary Table S2**. Thermal profile for the real-time PCR was amplification at 50°C for 2 min followed by 40 cycles at 95°C for 15 sec, 60°C for 30 sec and 72°C for 1 min. Melting curves were generated along with the mean Ct values and confirmed the generation of a specific PCR product. Healthy controls were used both as baseline references for relative quantification in RT-PCR (via the 2^-ΔΔCt method) and as a comparative group in statistical analyses. Cytokine expression levels and gene expression profiles were statistically compared between NSCLC patients and healthy controls to identify disease-associated alterations. Amplification of GAPDH was used as internal control for normalization. The results were expressed as fold change of control (Untreated samples (GAPDH)) using the 2^-ΔΔCT^ method. Each experiment was done in triplicates and repeated three times. Statistical significance was determined by Student’s t-test analysis (P<0.05) (9).

### Statistics analysis

Progression-Free Survival (PFS) was defined as the duration from the first day of immunotherapy to the date of disease progression, death, or last follow-up. Patients without progression or death were censored at the time of their last follow-up. Overall Survival (OS) was defined as the time from the first day of immunotherapy to death from any cause, with patients still alive at the last follow-up censored.

Logistic regression analysis was conducted to determine predictive factors associated with therapeutic response. The area under the curve (AUC) of the receiver operating characteristic (ROC) curves was calculated to assess the predictive accuracy for therapeutic response.

Univariate and multivariate Cox proportional hazards regression analyses were performed to identify cytokines significantly associated with progression-free survival (PFS) and overall survival (OS). The Cox model estimates the hazard ratio (HR) for each cytokine, reflecting the relative risk of an event as a function of its concentration as shown in Equation 1.


(1) $$\text{h}(\text{t}|\text{X})={\text{h}}_{0}\left(\text{t}\right)\text{exp}\left({\text{β}}_{1}{\text{X}}_{1}+{\text{β}}_{2}{\text{X}}_{2}+\ldots +{\text{β}}_{\text{p}}{\text{X}}_{\text{p}}\right)$$

where h(t∣X) is the hazard at time t, h_0_(t) is the baseline hazard, X_i_ are the covariates, and β_i_ are the regression coefficients estimated by partial likelihood. To validate the prognostic significance of cytokines identified from the Cox model and to determine optimal cutoff values for stratification, we subsequently conducted ROC analysis. Kaplan-Meier survival curves were generated to compare PFS and OS across different cytokine expression groups and the log-rank test was used to assess the statistical significance of differences between the survival distribution.

Variables with p<0.1 in univariate analysis were included in the multivariate analysis. Two-sided p-values < 0.05 were considered statistically significant. All statistical analyses were performed using Python, specifically the lifelines package (10).

## Results

### Clinical and pathological characteristics of the study cohort

A total of 64 patients undergoing immune checkpoint inhibitor (ICI) therapy were included in the study (**Table 1**). The age of participants ranged from 30 to 81 years, with a male predominance (65.6% males, n= 42, 34.3% females, n=22). Males had significantly higher mean height (172.02±6.19cm) and weight (71.90±5.83kg) compared to females (160.73±6.51cm and 65.55±4.61kg, respectively; *p*<0.0001 for both). However, mean BMI did not differ significantly between sexes (24.40±2.57 in males vs. 25.41±2.30 in females; *p*=0.1180). A vegetarian diet was predominant across the cohort (92.1%) with no significant difference between sexes.

**Table 1:** Comparison of clinical and demographic characteristics of non-small cell lung cancer patients’ characteristics between male and female patients (N = 64).

Variable	Male (Mean ± SD)	Female (Mean ± SD)	Male - Yes N (%)	Female -Yes N (%)	p-value
Age (years)	62.98 ± 9.21	65.32 ± 7.33			0.2726
**Height (cm)**	**172.02 ± 6.19 cm**	**160.73 ± 6.51 cm**			**0.0000**
**Weight (kg)**	**71.90 ± 5.83**	**65.55 ± 4.61**			**0.0000**
BMI	24.40 ± 2.57	25.41 ± 2.30			0.1180
Vegetarian Diet			39 (92.9%)	20 (90.9%)	1.0000
Diabetes Mellitus			22 (52.4%)	14 (63.6%)	0.5506
Hypertension			25 (59.5%)	15 (68.2%)	0.6835
Any Lung or Heart Disease			17 (40.5%)	10 (45.5%)	0.9072
**Smoking**			**20 (47.6%)**	**4 (18.2%)**	**0.0415**
**Alcohol Consumption**			**21 (50.0%)**	**0 (0.0%)**	**0.0002**
Tobacco Chewing			5 (11.9%)	0 (0.0%)	0.2320
Weight Loss			24 (57.1%)	15 (68.2%)	0.5552
Pleural Effusion			22 (52.4%)	16 (72.7%)	0.1915
Pre-treated Radiotherapy			10 (23.8%)	3 (13.6%)	0.5263
Pre-treated Chemotherapy			37 (88.1%)	22 (100.0%)	0.2320
Adenocarcinoma			18 (42.9%)	10 (45.5%)	1.0000
Squamous Cell Carcinoma			19 (45.2%)	12 (54.5%)	0.6568
Large Cell Carcinoma			4 (9.5%)	2 (9.1%)	1.0000
Metastasis			16 (38.1%)	10 (45.5%)	0.7631
Microsatellite Instability (MSS)			20 (47.6%)	11 (50.0%)	1.0000
Desmoplasia Collagen III (Present)			7 (16.7%)	2 (9.1%)	0.6531
TP53 Mutation (Present)			22 (52.4%)	6 (27.3%)	0.0973
Metastasis			16 (38.1%)	10(45.5%)	0.7631
Tumor Microenvironment Burden	10.43 ± 6.88	8.20 ± 8.34			0.2883

The Table presents the clinical and demographic characteristics of 64 patients diagnosed with non-small cell lung cancer. The data include continuous variables are presented as mean ± standard deviation (SD), and categorical variables are presented as counts and percentages (N (%)) of patients with the condition or attribute. The p-values for continuous variables were calculated using the independent two-sample *t*-test. For categorical variables, the p-values were obtained using the chi-square test. A p-value < 0.05 was considered statistically significant.

Comorbid conditions such as diabetes mellitus (56.2%) and hypertension (62.5%) were common. Smoking (47.6% males compared to 18.2% females; *p*=0.0415) and alcohol consumption (50.0% males vs. 0% females; *p*=0.0002) were significantly more prevalent among males. Tobacco chewing was reported by 7.8% of the overall cohort. No significant differences were found between males and females with regard to the prevalence of lung or heart diseases (57.8%), weight loss, pleural effusion (59.3%), or prior cancer treatments including radiotherapy (20.3%) and chemotherapy (92.1%).

Histological analysis showed squamous cell carcinoma (48.4%) and adenocarcinoma (43.7%) as the most frequent subtypes, with no significant gender-based distribution. TP53 mutations were more common in males (52.4%) compared to females (27.3%), though the difference was not statistically significant (*p*=0.0973). Similarly, metastasis was slightly more common in females (45.5%) than in males (38.1%), but this difference was not statistically significant (*p*=0.7631). The tumor microenvironment burden ranged from 0.72 to 25 and microsatellite instability analysis revealed Low MSI in 51.5% and Stable MSI in 48.4% of cases. Desmoplasia with collagen III expression was observed in 14% of patients, while 85.9% showed no evidence of desmoplasia.

### Association between cytokine expression with Response to checkpoint inhibitor therapy in NSCLC patients

To explore circulating non-invasive biomarkers for predicting response to immune checkpoint inhibitor (ICI) therapy in cancer, in depth cytokine profiling was conducted using multiplex Luminex –Flex Map 3D platform. Blood samples were collected at baseline. The panel included 15 cytokines and immune-related factors **i**ncluding interleukins (ILs), interferons (IFNs), tumor necrosis factor (TNF) superfamily members, colony stimulating factors (CSF), chemokines, and growth factors (GFs). These molecules are secreted by immune cells such as monocytes, macrophages, T cells, B cells, and NK cells as well as by certain non-immune cells, including endothelial cells, epidermal cells, and fibroblasts. The cytokines analyzed were: IL-1α, IL-1β, IL-2, IL-4, IL-10, IL-13, IL-15, IL-17, IL-18, IL-21, IL-23, GM-CSF, TNFα, IFN- γ and sPD-L1.

**Figure 1:**
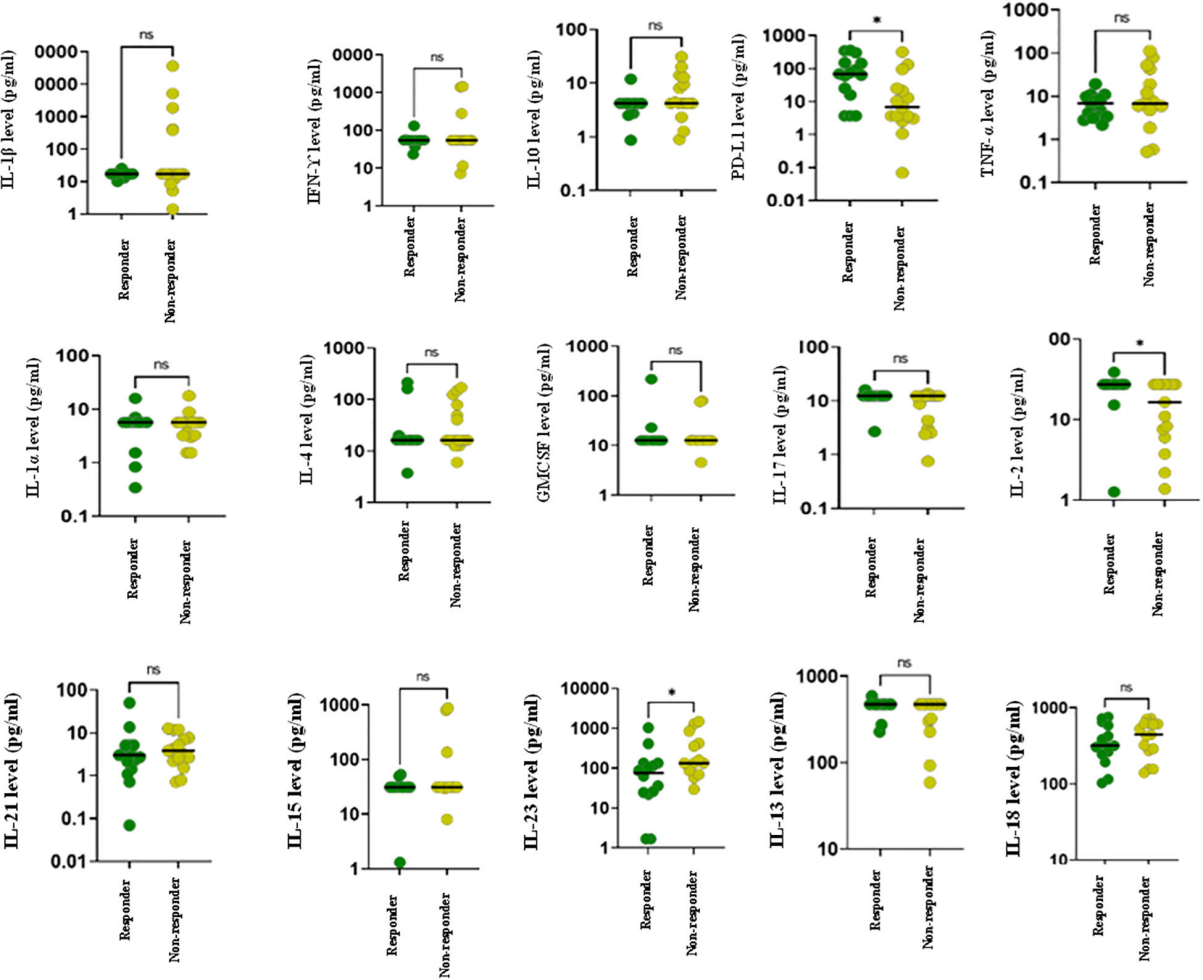
Cytokine profiling of advanced lung cancer patients receiving ICI therapy at baseline using Luminex. The figure represents dot plots showing baseline cytokine levels for various cytokines in responders (green) and non-responders (yellow) to ICI treatment. Each subplot represents a distinct cytokine, with individual data points corresponding to patient measurements. Horizontal lines within each plot indicate the median cytokine levels for each group. Statistically significant differences between groups are denoted by *.

The longitudinal comparison of responders and non-responders was performed using Wilcoxon rank sum analysis. The results revealed that at the baseline, sPDL-1, IL-2 and IL-23 levels were significantly elevated in responders as compared to non-responders (p<0.05). No statistically significant differences were observed for the remaining cytokines between the two groups (**Figure 1**).

**Figure 2:**
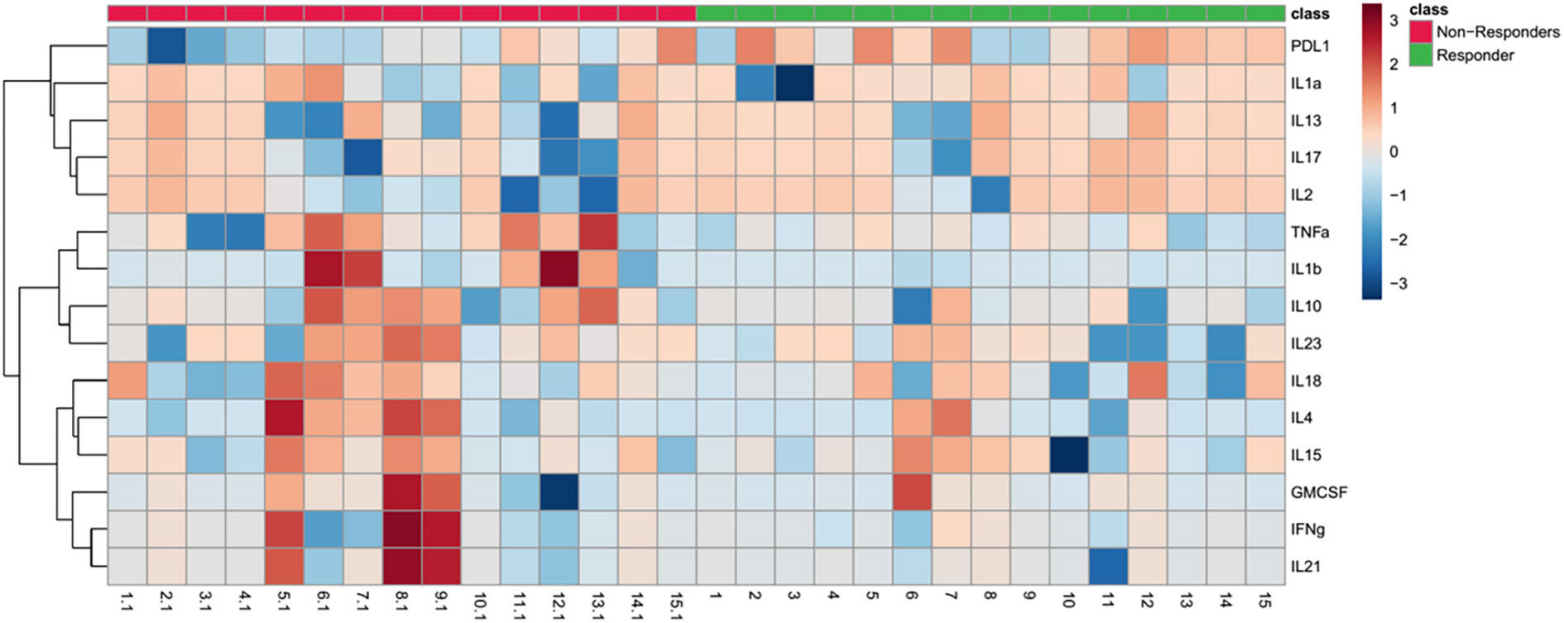
Heat map depicting baseline cytokine profiles in responders and non-responders to ICI therapy. The heatmap illustrates the expression levels of 15 cytokines at baseline in responders (green) and non-responders (red). The color scale reflects cytokine expression levels, with red indicating higher levels and blue indicating lower levels. The dendrogram on the left clusters cytokines based on similarities in their expression patterns across subjects, highlighting potential clustering of immune response profiles.

In concordant with these findings, **Figure 2** presents a heat map comparing the baseline levels of 15 cytokines between responders and non-responders. The data clearly highlight a notable upregulation of soluble PD-L1 (sPD-L1) in responders at baseline.

**Figure 3:**
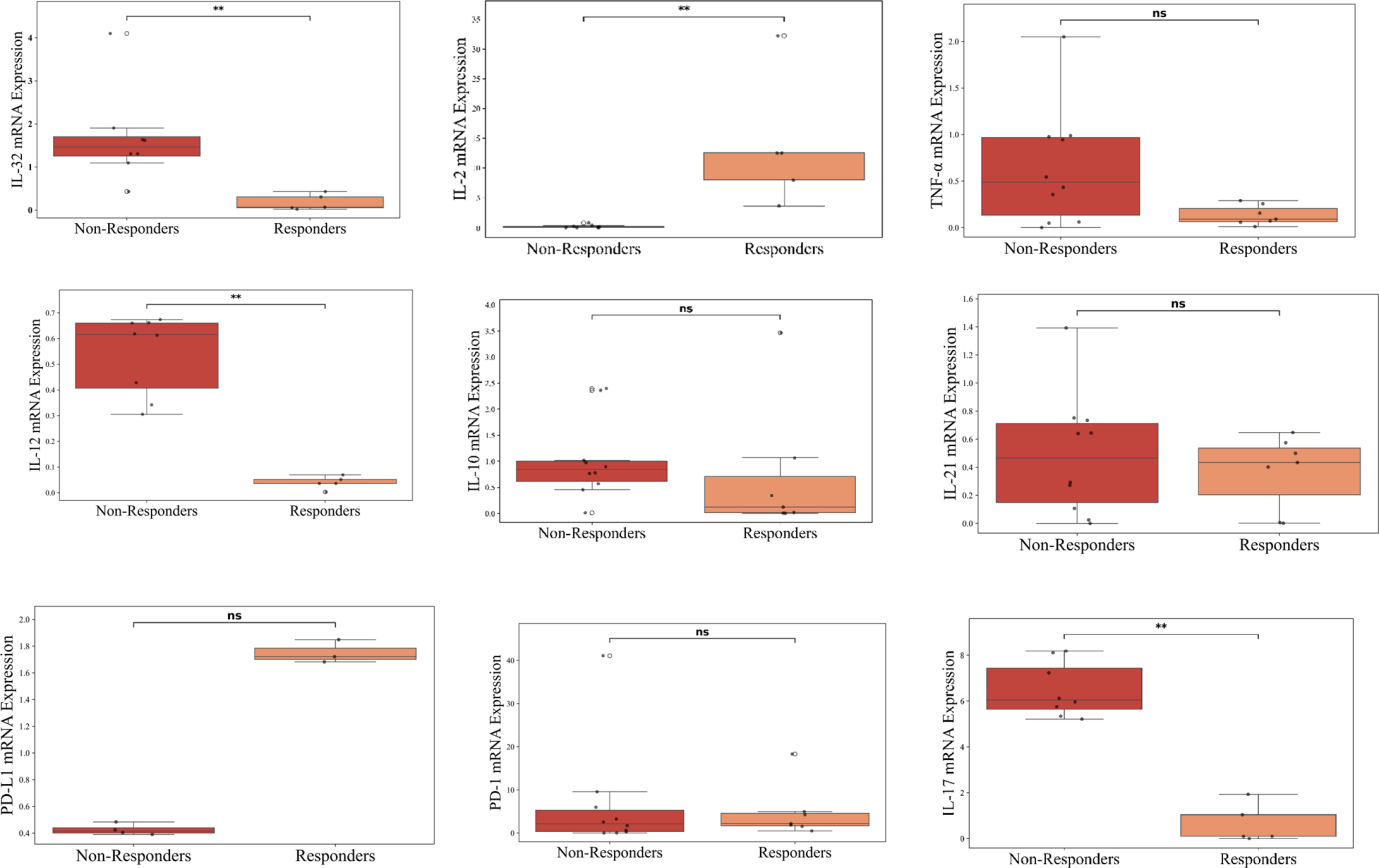
Cytokine profiling of advanced lung cancer patients receiving ICI therapy at baseline using RT-PCR. The figure represents RT-PCR-based gene expression analysis conducted on a subset of 17 patients (Responders = 7, Non-Responders = 10) and 3 Healthy Controls; n=3. The figure represents box plots illustrating the comparison of baseline cytokine levels between non-responders (red) and responders (orange) (normalized to Healthy Controls) to ICI therapy. Each box represents the interquartile range, with the horizontal line indicating the median value, and the whiskers extending to the minimum and maximum values within 1.5 times the interquartile range. Individual data points are plotted to show variability within each group. Statistically significant differences are marked with asterisks (**p < 0.01), while “ns” denotes non-significant comparisons.

To validate cytokine expression at the transcript level, RT-PCR was performed on PBMCs isolated from patients with NSCLC. Here, RT-PCR-based gene expression analysis was conducted on a subset of 17 patients (Responders = 7, Non-Responders = 10) and 3 Healthy Controls. Gene expression analysis revealed distinct transcriptional patterns between responders and non-responders to ICI therapy. Importantly, IL-2 expression was significantly upregulated (~10 folds) in responders compared to non-responders (p < 0.01), supporting its role as potential predictive biomarker for positive treatment outcomes. In contrast, IL-32, IL-12 and IL-17 showed elevated expression in non-responders (p < 0.01), suggesting a potential association with immune suppression and resistance to ICI therapy as shown in **Figure 3**.

### Association of cytokines with response status in NSCLC patients undergoing ICI therapy

To explore the relationship between cytokine levels and treatment response, we performed logistic regression analysis on plasma samples collected prior to immune checkpoint inhibitor (ICI) therapy (**Supplementary Table S3**).

**Figure 4:**
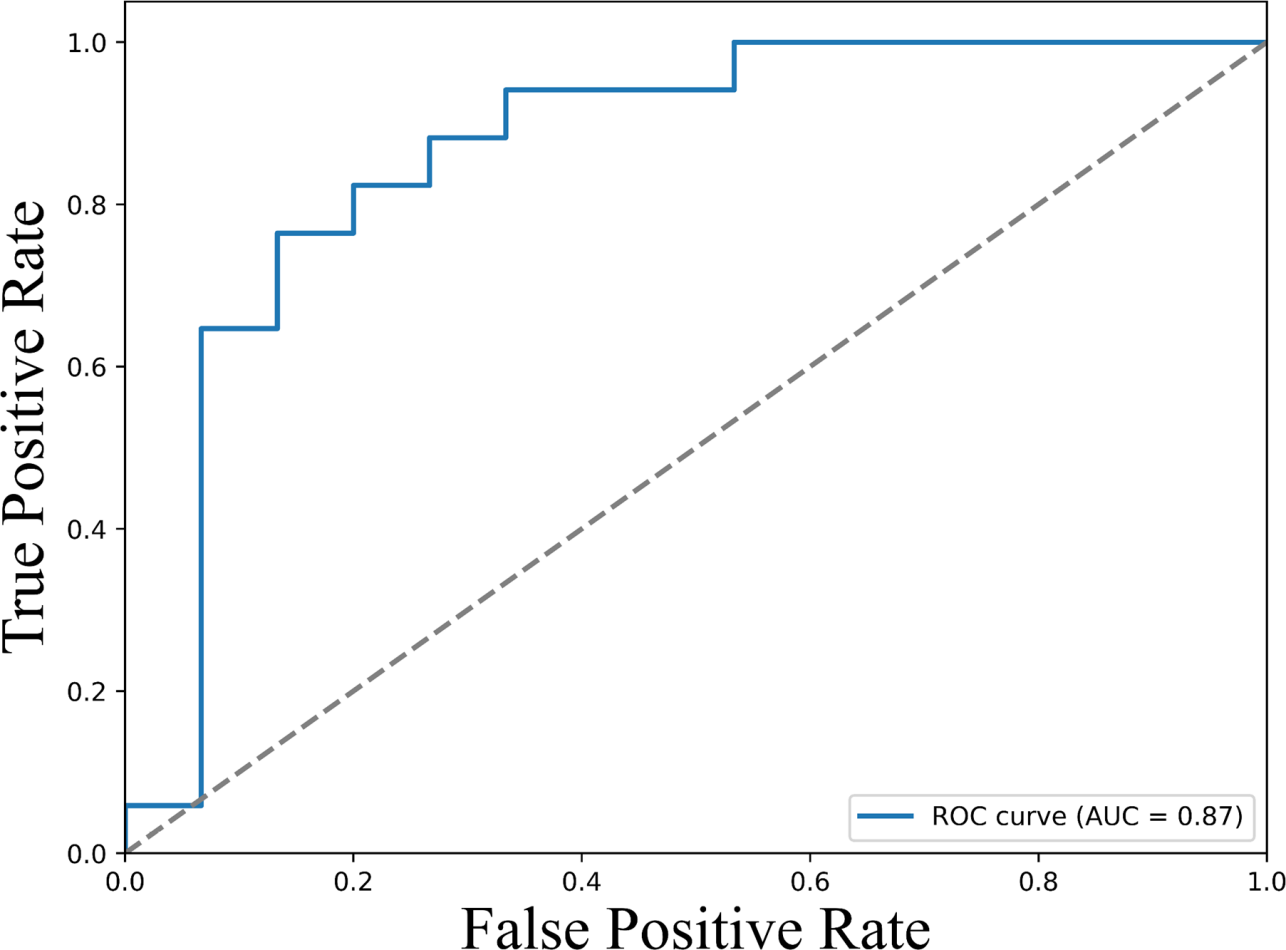
ROC curves illustrating the predictive value of baseline sPD-L1 levels for therapeutic response. The ROC curve displays the true positive rate (sensitivity) versus the false positive rate (1 - specificity) across a range of sPD-L1 threshold values. The area under the curve (AUC) is 0.87 (95% CI: 0.76–0.96), demonstrating strong discriminatory power of sPD-L1 as a biomarker for predicting clinical response to immune checkpoint inhibitor therapy. Higher AUC values indicate greater predictive accuracy.

Analysis using univariate logistic regression identified TNF-α, PDL-1, IL-10, IL-17, IL-2, IL-13, IL-23 responsive to ICI therapy (**Supplementary Table S3**). Further analysis using multivariate logistic regression identified sPD-L1 (Odd Ratio of 1.51 (95% CI: 1.046-2.81, p <0.005)), as a potential predictive biomarker for response to ICI therapy (**Table 2**). To assess the predictive performance of cytokine levels, ROC curve analysis was performed. The analysis yielded an area under the curve (AUC) of 0.87 (95% CI: 0.76–0.96) (**Figure 4**), indicating a high level of diagnostic accuracy for predicting treatment response.

**Table 2:** Association between cytokine levels and response status by multivariate analysis.

Cytokine	Odd ratio (95% CI)	p-value
PD-L1	1.51(1.046-2.81)	<0.005

Multivariate logistic regression analysis showing the association between cytokine expression levels and response status. PD-L1 showed a odd ratio (HR) of 1.51 (95% CI: 1.046-2.81, p <0.005, indicating a strong predictive effect on treatment response.

### Association of cytokines with survival outcomes in NSCLC patients undergoing ICI therapy

Univariate Cox regression analysis was performed to evaluate the association of baseline cytokine levels and clinical variables with progression-free survival (PFS) and overall survival (OS) as shown in **Supplementary Table S4** and **Supplementary Table S5**. Variables demonstrating statistical significance (p < 0.05) in the univariate analysis were subsequently included in the multivariate Cox regression model to identify independent predictors of survival outcomes.

For progression-free survival (PFS), multivariate Cox regression analysis identified IL-2 (HR = 0.67, 95% CI: 0.58–0.79, p = <0.005), sPD-L1 (HR = 0.15, 95% CI: 0.05–0.48, p = <0.005), and IL-23 (HR = 1.18, 95% CI: 1.13–0.98, p = <0.005) as independent predictors of outcome (**Table 3**).

**Table 3:** Association of baseline cytokine levels and clinical variables with progression-free survival (PFS) by multivariate analysis.

Cytokine	Hazard ratio (95% CI)	p-value
IL-2	0.67(0.58-0.79)	<0.005
PD-L1	0.15(0.05-0.43)	<0.005
IL-23	1.18(1.13-1.24)	<0.005

Multivariate Cox regression analysis showing the association between progression-free survival (PFS) and cytokine expression levels. IL-2 exhibits a hazard ratio (HR) of 0.67 (95% CI: 0.58 – 0.79) with a p-value < 0.005, indicating a significant association with reduced mortality risk. Similarly, sPD-L1 has an HR of 0.15 (95% CI: 0.05 – 0.43) and p-value of <0.005, associated with increased risk of progression. In contrast, IL-23 shows an HR of 1.18 (95% CI: 1.13 – 1.24) with a p-value < 0.005, indicating a significant association with increased mortality risk.

Similarly, for overall survival (OS), multivariate analysis revealed that IL-2 (HR = 0.63, 95% CI: 0.54-0.75, p = <0.005), sPD-L1 (HR = 0.29, 95% CI: 0.11–0.80, p = 0.002) and IL-23 (HR = 1.08, 95% CI: 1.03–0.1.13, p = <0.005) were significantly associated with survival outcomes (**Table 4**).

**Table 4:** Association of baseline cytokine levels and clinical variables with overall survival (OS) by multivariate analysis.

Cytokine	Hazard ratio (95% CI)	p-value
IL-2	0.63(0.54-0.75)	<0.005
PD-L1	0.29(0.11-0.80)	0.02
IL-23	1.08(1.03-1.13)	<0.005

Multivariate Cox regression analysis showing the association between overall survival (OS) and cytokine expression levels. IL-2 exhibits a hazard ratio (HR) of 0.63 (95% CI: 0.54 – 0.75) with a p-value < 0.005, indicating a significant association with reduced mortality risk. sPD-L1 has an HR of 0.29 (95% CI: 0.11 – 0.80) and p-value of 0.02, suggesting a strong protective effect on survival. In contrast, IL-23 shows an HR of 1.08 (95% CI: 1.03 – 1.13) with p-value < 0.005, indicating a significant association with increased mortality risk.

**Figure 5:**
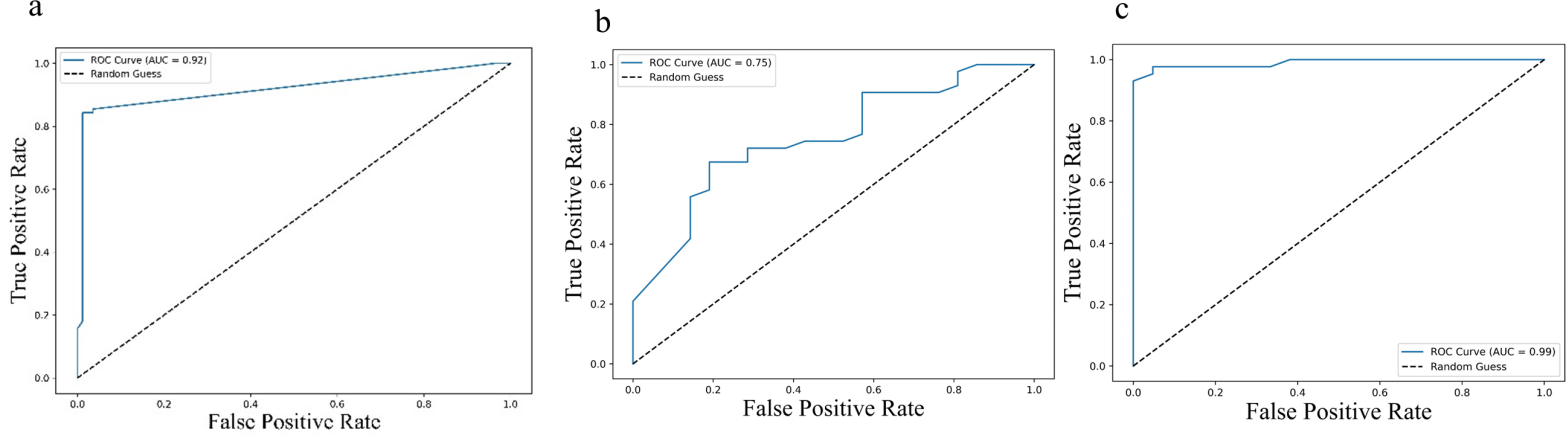
ROC curves illustrating predictive performance of cytokine levels in relation to progression-free survival (PFS): **(a)** IL-2, **(b)** IL-23, and **(c)** PD-L1. Each curve plots the true positive rate against the false positive rate across various thresholds. The area under the curve (AUC) values were 0.92 (95% CI: 0.89–0.95) for IL-2, 0.75 (95% CI: 0.69–0.81) for IL-23, and 0.99 (95% CI: 0.98–1.00) for PD-L1, indicating their respective discriminatory capacities. Higher AUC values reflect stronger predictive power for PFS.

**Figure 6:**
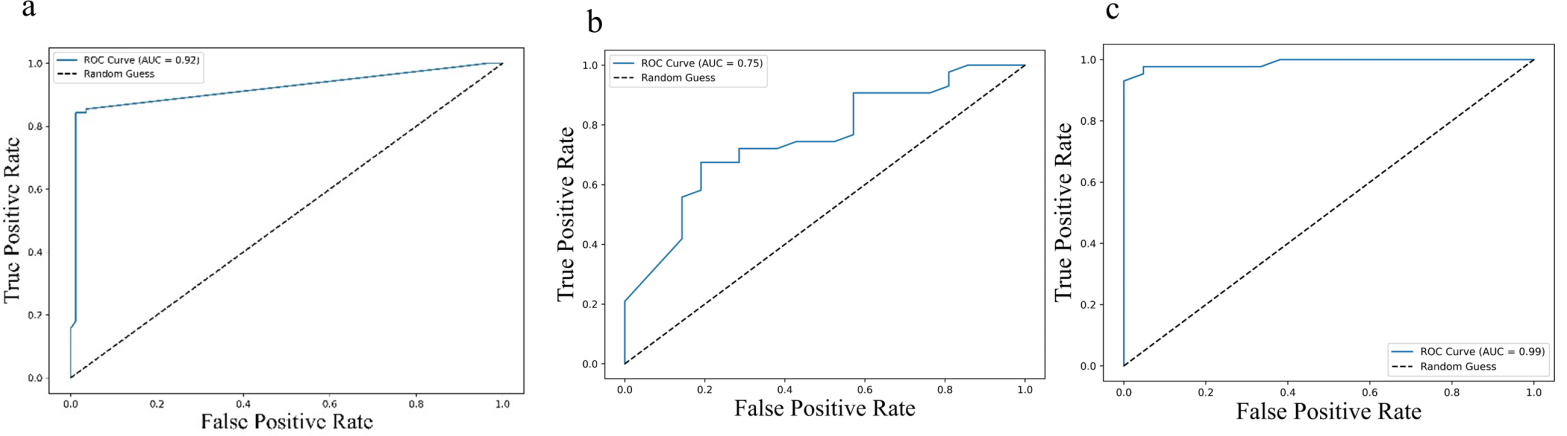
Receiver Operating Characteristic (ROC) curves illustrating predictive value of cytokine levels for overall survival (OS): **(a)** IL-2, **(b)** IL-23, and **(c)** PD-L1. Each curve plots the true positive rate against the false positive rate across various thresholds. The area under the curve (AUC) values—0.92 (95% CI: 0.89–0.95) for IL-2, 0.75 (95% CI: 0.69–0.81) for IL-23, and 0.99 (95% CI: 0.98–1.00) for PD-L1—highlight the varying discriminatory capacities of these cytokines to predict OS, with higher AUCs indicating greater predictive accuracy.

Further, Receiver operating characteristic (ROC) analysis was performed for the cytokines identified significant in the multivariate Cox regression. For progression-free survival (PFS), the area under the curve (AUC) values were 0.92 (95% CI: 0.89 – 0.95) for IL-2, 0.75 (95% CI: 0.69 – 0.81) for sPD-L1, and 0.99 (95% CI: 0.98 – 1.00) for IL-23, indicating strong predictive performance (**Figure 5**). Similar trends were observed for overall survival (OS), with AUC values of 0.92 (95% CI: 0.89 – 0.95) for IL-2, 0.75 (95% CI: 0.69 – 0.81) PD-L1 and 0.99 (95% CI: 0.98 – 1.00) for IL-23 (**Figure 6**), further supporting their potential as robust prognostic biomarkers.

**Figure 7:**
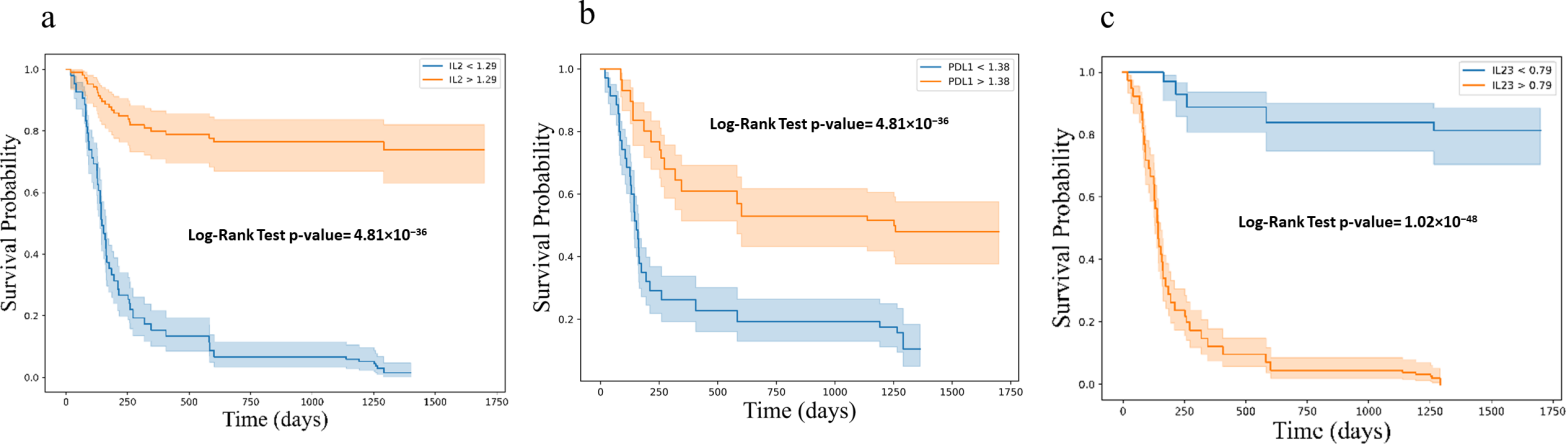
Kaplan-Meier survival curves for progression free survival (PFS) based on cytokine levels **(a)** IL2 **(b)** PD-L1 and **(c)** IL23, showing stratification into high and low expression groups. Survival differences were assessed using the log-rank test. Kaplan-Meier survival curves illustrating progression-free survival (PFS) stratified by cytokine expression levels: **(a)** IL-2, **(b)** PD-L1, and **(c)** IL-23. Each plot categorizes patients into high (orange) and low (blue) expression groups based on cytokine levels, demonstrating distinct survival probabilities over time. The log-rank test p-values for each cytokine indicate highly significant differences in PFS between the groups.

**Figure 8:**
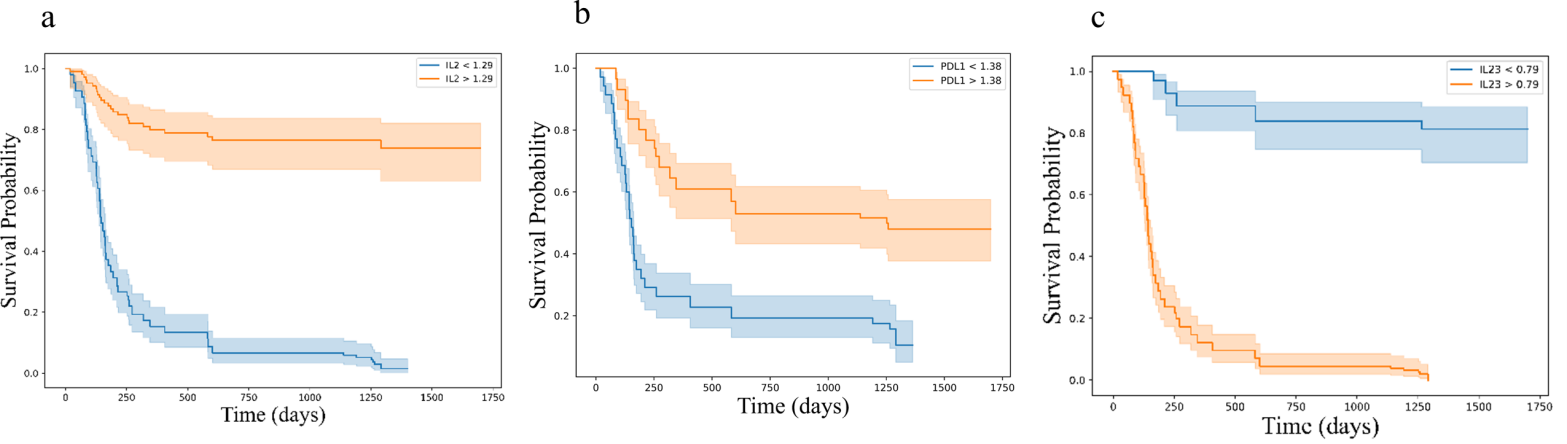
Kaplan-Meier survival curves for overall survival (OS) based on cytokine levels **(a)** IL2 **(b)** PD-L1 and **(c)** IL23, showing stratification into high and low expression groups. Survival differences were assessed using the log-rank test. Kaplan-Meier survival curves illustrating overall survival (OS) stratified by cytokine expression levels: **(a)** IL-2, **(b)** PD-L1, and **(c)** IL-23. Each plot categorizes patients into high (orange) and low (blue) expression groups based on cytokine levels, demonstrating distinct survival probabilities over time. The log-rank test p-values for each cytokine indicate highly significant differences in PFS between the groups.

Kaplan-Meier survival analysis, stratified by cytokine expression levels using ROC-determined cut-offs, further confirmed these associations. For progression-free survival (PFS), higher baseline levels of IL-2 (log-rank p < 0.001) and sPD-L1 (log-rank p < 0.001), along with lower levels of IL-23 (log-rank p < 0.001) were significantly associated with improved outcomes (**Figure 7**). Similar associations were observed for overall survival (OS), where higher levels of IL-2 (log-rank p < 0.001) and sPD-L1 (log-rank p < 0.001), as well as lower levels of IL-23 (log-rank p < 0.001) were predictive of longer survival (**Figure 8**).

Combined analysis of IL-2 and PD-L1 revealed complementary predictive power for both progression-free survival (PFS) (HR for IL-2 = 0.86 (95% CI: 0.79 – 0.93) with a p-value = 0.0003, PD-L1 = 0.14 (95% CI: 0.09 – 0.21) with a p-value < 0.0001) and overall survival (OS) (HR for IL-2 = 0.83 (95% CI: 0.76 – 0.91) with a p-value < 0.0001, PD-L1 = 0.15 (95% CI: 0.10 – 0.22) with a p-value < 0.0001). Cox regression analysis confirmed the combined prognostic significance of these cytokines (IL-2, HR= 0.8, PD-L1, HR= 0.15) exhibiting a substantially reduced hazard ratio for both disease progression and mortality (**Tables 5**, **Table 6**).

**Table 5:** Multivariate Cox regression analysis considering both IL-2 and PD-L1 together with PFS in relation to survival outcomes.

Cytokine	Hazard ratio (95% CI)	p-value
Combination		
IL-2 PD-L1	0.86 (0.79–0.93) 0.14 (0.09–0.21)	0.0003< 0.0001

Multivariate Cox regression analysis evaluating the combined impact of IL-2 and PD-L1 expression levels on progression free survival (PFS) in relation to survival outcomes. The hazard ratio (HR) for IL-2 is 0.86 (95% CI: 0.79 – 0.93) with a p-value 0.0003, indicating that higher IL-2 levels are significantly associated with reduced risk of mortality. The HR for PD-L1 is 0.14 (95% CI: 0.09 – 0.21) with a p-value < 0.0001, demonstrating a strong protective effect of higher PD-L1 expression on survival.

**Table 6:** Multivariate Cox regression analysis considering both IL-2 and PD-L1 together with OS in relation to survival outcomes.

Cytokine	Hazard ratio (95% CI)	p-value
Combination		
IL-2 PD-L1	0.83(0.76 – 0.91) 0.15(0.10 – 0.22)	< 0.0001< 0.0001

Multivariate Cox regression analysis evaluating the combined impact of IL-2 and PD-L1 expression levels on overall survival (OS) in relation to survival outcomes. The hazard ratio (HR) for IL-2 is 0.83 (95% CI: 0.76 – 0.91) with a p-value < 0.0001, indicating that higher IL-2 levels are significantly associated with reduced risk of mortality. The HR for PD-L1 is 0.15 (95% CI: 0.10 – 0.22) with a p-value < 0.0001, demonstrating a strong protective effect of higher PD-L1 expression on survival.

**Figure 9:**
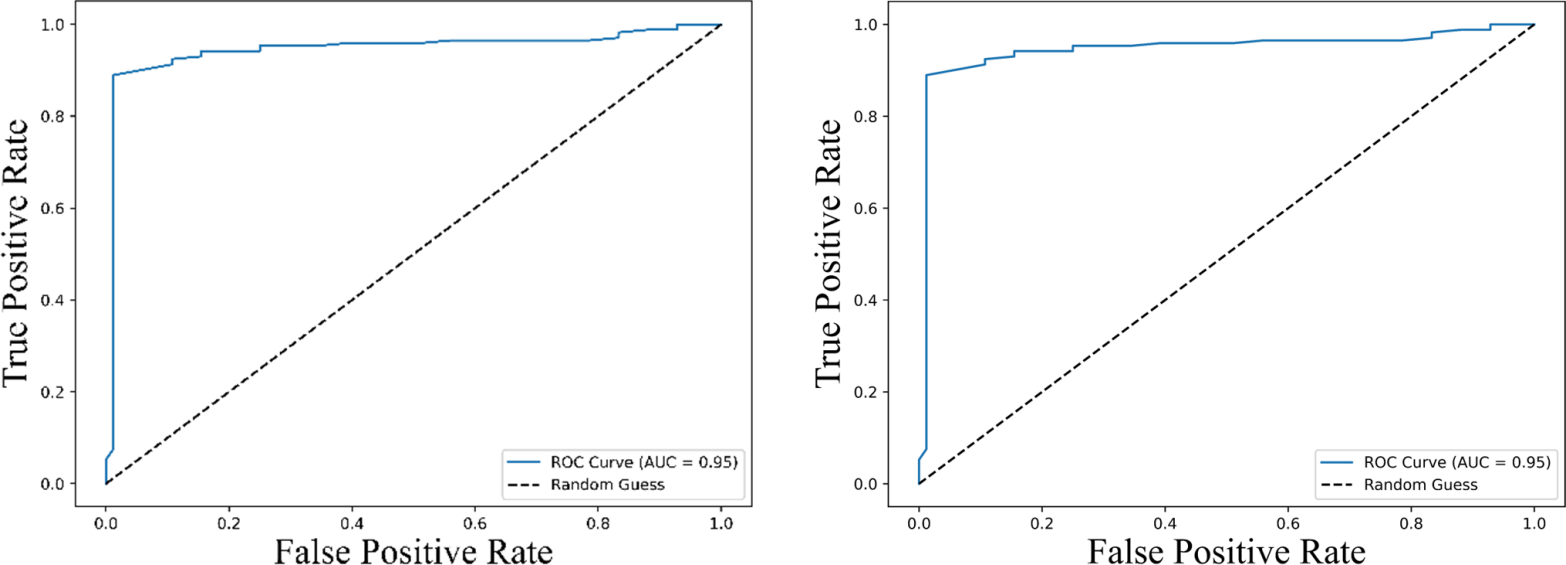
Receiver Operating Characteristic (ROC) curves illustrating the predictive performance of the combined expression levels of IL-2 and PD-L1 for survival outcomes. The left panel represents the ROC curve for predicting progression-free survival (PFS), while the right panel shows the ROC curve for overall survival (OS). Both curves achieve an area under the curve (AUC) of 0.95 (95% CI: 0.89 – 1.00), indicating excellent predictive accuracy. The dashed line represents a random guess (AUC = 0.5), highlighting the superior discriminatory ability of the combined biomarker model.

Receiver operating characteristic (ROC) curve analysis of the combined IL-2 and PD-L1 levels further strengthened their predictive power, showing an improved AUC (~ 0.95) (**Figure 9**) compared to either cytokine alone, demonstrating the enhanced discriminatory capacity of the combined biomarker model for survival prediction.

**Figure 10:**
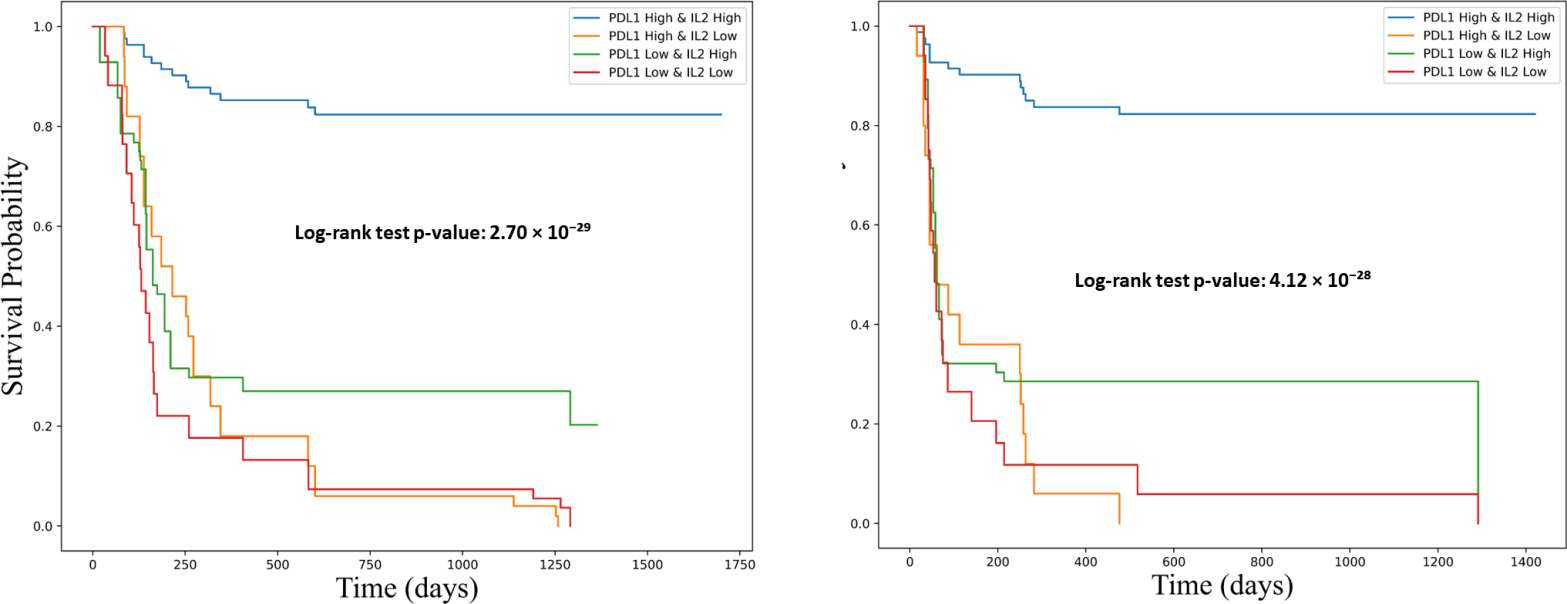
Kaplan-Meier survival curves showing the association of combined PD-L1 and IL-2 expression levels with overall survival (OS) and progression-free survival (PFS). Survival differences were assessed using the log-rank test. The left panel illustrates progression-free survival (PFS), and the right panel shows overall survival (OS). Patients were stratified into four groups based on PD-L1 and IL-2 expression: PD-L1^high/IL-2^high (blue), PD-L1^high/IL-2^low (orange), PD-L1^low/IL-2^high (green), and PD-L1^low/IL-2^low (red). Both survival analyses reveal significantly improved outcomes in the PD-L1^high/IL-2^high group. The log-rank test p-values were 4.12 × 10^−28^ for PFS and 2.70 × 10^−29^ for OS, underscoring a strong association between combined biomarker expression and favorable clinical outcomes.

To further explore the clinical relevance of this combined biomarker model, patients were stratified into four groups based on their IL-2 and sPD-L1 expression levels: Group 1 (high IL-2/high sPD-L1), Group 2 (low IL-2/high sPD-L1), Group 3 (high IL-2/low sPD-L1), and Group 4 (low IL-2/low sPD-L1). Kaplan–Meier survival analysis demonstrated that patients in Group 1 had significantly improved PFS and OS compared to the other groups (log-rank p < 0.001), highlighting the synergistic effect of IL-2 and sPD-L1 on survival outcomes (**Figure 10**).

## Conclusion

This study highlights the prognostic and predictive value of circulating cytokines particularly IL-2, sPD-L1, and IL-23 in patients with advanced non-small cell lung cancer (NSCLC) undergoing immune checkpoint inhibitor (ICI) therapy. Elevated baseline levels of IL-2 and sPD-L1 were associated with improved clinical responses and prolonged progression-free and overall survival, while increase IL-23 levels correlated with poorer outcomes, suggesting its potential role in immune suppression and resistance to ICIs. Notably, the combined assessment of IL-2 and sPD-L1 enhanced predictive accuracy, emphasizing the advantage of multi-marker approaches for more precise patient stratification. These findings, validated through both multiplex cytokine analysis and RT-PCR in PBMCs, highlight the value of integrating immune profiling into routine clinical practice to enable more personalized and effective immunotherapy strategies. Future large-scale, multi-center studies are essential to validate these biomarkers and establish standardized protocols for their clinical application.

## Discussion

In this study, we investigated the cytokine profiling of patients with non-small cell lung cancer (NSCLC) undergoing immune checkpoint inhibitor (ICI) therapy, aiming to assess potential biomarkers associated with treatment response and survival outcomes. Our findings reveal distinct cytokine expression patterns between responders and non-responders, suggesting their potential role in predicting treatment outcomes.

A key observation was the differential expression of pro-inflammatory and immunoregulatory cytokines among responders and non-responders. Elevated levels of IL-2 and PDL-1 were significantly associated with favorable clinical responses to ICI therapy, consistent with previous studies linking robust anti-tumor immune response to cytokine-mediated T-cell activation. Conversely, non-responders exhibited increased levels of IL-23 which are known to contribute to an immunosuppressive tumor microenvironment, thereby potentially attenuating the efficacy of ICIs.

IL-2, a well-characterized cytokine has been extensively studied for its dual role in promoting immune responses and maintaining immune tolerance. Recent studies have explored its applications in autoimmune disorders and cancer immunotherapy. Lykhopiy et al. (2023) highlighted the role of IL-2 in regulating T cells, particularly its function in modulating regulatory T cells (Tregs) to maintain immune homeostasis in autoimmune conditions such as systemic lupus erythematosus and type 1 diabetes (11). IL-2 has also been recognized for its ability to stimulate cytotoxic T lymphocytes (CTLs) and natural killer (NK) cells, which are critical in antitumor immunity. Rokade et al. (2024) emphasized the development of IL-2-based therapies that enhance therapeutic efficacy while mitigating toxicity (12). Similarly, Kim et al. (2021) explored how IL-2 and IL-7 support T-cell proliferation and survival, thereby enhancing antitumor responses (13). Xu et al. (2022) found that interventions such as microwave ablation in NSCLC patients can alter systemic cytokine profiles particularly IL-2 and IFN-γ, indicating a shift in immune status post-treatment (14). Mao et al. (2022) further confirmed, through a meta-analysis, that elevated IL-2 levels are associated with improved outcomes in patients receiving ICIs, reinforcing the utility of cytokine monitoring as a predictive tool (15).

Our study also identified high level of sPD-L1 as a significant plasma biomarker, aligning with findings by Shimizu et al. (2023), who reported that sPD-L1 levels dynamically reflect treatment response in patients receiving PD-1 inhibitors (16). Similarly, Ancel et al. (2023) and Machiraju et al. (2021) highlighted the role of soluble immune checkpoints and cytokines in predicting ICI efficacy and resistance, further supporting our observations (17, 18).

IL-23, in contrast, appears to contribute to immune suppression within the tumor microenvironment. The IL-23/IL-17 axis plays a significant role in immune regulation, particularly in autoimmunity and cancer immunotherapy. Li et al. (2024) reported successful management of pre-existing psoriatic arthritis in cancer patients undergoing immune checkpoint inhibitor (ICI) therapy through targeting this axis. This suggests that modulating IL-23 and IL-17 pathways could mitigate immune-related adverse events (irAEs) while preserving anti-tumor immunity (19).

The IL-23/IL-17 axis is increasingly recognized for its role in cancer immunopathology. Wertheimer et al. (2024) demonstrated that IL-23 supports the stability of an effector Treg phenotype within tumors, potentially undermining antitumor immunity (20). Li et al. (2024) further showed that targeting this axis can manage immune-related adverse events in patients undergoing ICI therapy (19). Liu et al. (2020) underscored the prognostic significance of IL-23 and Th17 cytokines in NSCLC, proposing them as potential markers for disease progression and immune modulation (21).

To further enhance the predictive utility of cytokines, our combined analysis of IL-2 and sPD-L1 demonstrated superior prognostic performance compared to individual markers. Kaplan–Meier analysis of patients stratified by combined cytokine profiles revealed that those with high IL-2 and sPD-L1 levels experienced significantly longer progression-free and overall survival. These findings emphasize the added value of multiplex biomarker models in refining patient stratification and optimizing treatment decisions.

Despite these promising insights, this study has certain limitations. The relatively small sample size and single-institutional design may limit the generalizability of our findings. Recruiting a cohort of 64 NSCLC patients treated with anti-PD-1 checkpoint inhibitors is challenging, given the heterogeneity of this patient population. Another limitation of this study is that no formal modeling approaches such as regularization, multicollinearity assessment, or cross-validation were applied, primarily due to the limited sample size. This may restrict the generalizability of the findings. Additionally, cytokine levels were measured at discrete time points, which may not fully reflect dynamic fluctuations throughout treatment. Future studies involving larger, multi-center cohorts with longitudinal sampling are needed to validate these findings and establish standardized cytokine-based predictive models.

In conclusion, our study underscores the potential utility of cytokine profiling, particularly IL-2, sPD-L1, and IL-23 into clinical practice to improve patient selection and personalize immunotherapy strategies in advanced NSCLC. These biomarkers may not only enhance treatment efficacy but also help anticipate resistance and toxicity. Further research is warranted to refine these biomarkers and explore their applicability in broader patient populations.

## Data Availability

The datasets presented in this study can be found in online repositories. The names of the repository/repositories and accession number(s) can be found in the article/**Supplementary Material**.

## References

[1] Franceschi S Bidoli E. The epidemiology of lung cancer. Ann Oncol. (1999) 10:S3–6. doi: 10.1093/annonc/10.suppl_5.S3, PMID: 10582131

[2] Cullen M. Lung cancer • 4: Chemotherapy for non-small cell lung cancer: the end of the beginning. Thorax. (2003) 58:352–6. doi: 10.1136/thorax.58.4.352, PMID: 12668803 PMC1746634

[3] Sacher AG Gandhi L. Biomarkers for the clinical use of PD-1/PD-L1 inhibitors in non–small-cell lung cancer: a review. JAMA Oncol. (2016) 2:1217–22. doi: 10.1001/jamaoncol.2016.0639, PMID: 27310809

[4] Scartozzi M Franciosi V Campanini N Benedetti G Barbieri F Rossi G. Mismatch repair system (MMR) status correlates with response and survival in non-small cell lung cancer (NSCLC) patients. Lung Cancer. (2006) 53:103–9. doi: 10.1016/j.lungcan.2006.03.008, PMID: 16716446

[5] Boumber Y. Tumor mutational burden (TMB) as a biomarker of response to immunotherapy in small cell lung cancer. J Thorac Dis. (2018) 10:4689–93. doi: 10.21037/jtd.2018.07.120, PMID: 30233840 PMC6129910

[6] Garon EB Rizvi NA Hui R Leighl N Balmanoukian AS Eder JP. Pembrolizumab for the treatment of non–small-cell lung cancer. N Engl J Med. (2015) 372:2018–28. doi: 10.1056/NEJMoa1501824, PMID: 25891174

[7] Matanić D Beg-Zec Z Stojanović D Matakorić N Flego V Milevoj-Ribić F. Cytokines in patients with lung cancer. Scand J Immunol. (2003) 57:173–8. doi: 10.1046/j.1365-3083.2003.01205.x, PMID: 12588664

[8] Liu Y Gao Y Lin T. Expression of interleukin-1 (IL-1), IL-6, and tumor necrosis factor-α (TNF-α) in non-small cell lung cancer and its relationship with the occurrence and prognosis of cancer pain. Ann Palliat Med. (2021) 10:12759–66. doi: 10.21037/apm-21-3471, PMID: 35016421

[9] Chang DH Rutledge JR Patel AA Heerdt BG Augenlicht LH Korst RJ. The effect of lung cancer on cytokine expression in peripheral blood mononuclear cells. PloS One. (2013) 8:e64456. doi: 10.1371/journal.pone.0064456, PMID: 23762239 PMC3675097

[10] Sui X Jiang L Teng H Mi L Li B Shi A. Prediction of clinical outcome in locally advanced non-small cell lung cancer patients treated with chemoradiotherapy by plasma markers. Front Oncol. (2021) 10:625911. doi: 10.3389/fonc.2020.625911, PMID: 33680949 PMC7925829

[11] Lykhopiy V Malviya V Humblet-Baron S Schlenner SM Dewaele M Liston A. IL-2 immunotherapy for targeting regulatory T cells in autoimmunity. Genes Immun. (2023) 24:248–62. doi: 10.1038/s41435-023-00221-y, PMID: 37741949 PMC10575774

[12] Rokade S Damani AM Oft M Emmerich J. IL-2 based cancer immunotherapies: an evolving paradigm. Front Immunol. (2024) 15:1433989. doi: 10.3389/fimmu.2024.1433989, PMID: 39114660 PMC11303236

[13] Kim JH Lee KJ Lee SW. Cancer immunotherapy with T-cell targeting cytokines: IL-2 and IL-7. BMB Rep. (2021) 54:21–30. doi: 10.5483/BMBRep.2021.54.1.257, PMID: 33407991 PMC7851446

[14] Xu H Tan X Kong Y Huang Y Wei Z Ye X. Microwave ablation of non-small cell lung cancer tumors changes plasma levels of cytokines IL-2 and IFN-γ. J Cancer Res Ther. (2022) 18:532–44. doi: 10.4103/jcrt.jcrt_211_22, PMID: 35645125

[15] Mao Y Huang Y Wang M Xu L Wang L. Peripheral cytokines and immune checkpoint inhibitors-related outcomes in patients with cancer: a systematic review and meta-analysis. Front Immunol. (2022) 13:884592. doi: 10.3389/fimmu.2022.884592, PMID: 36072577 PMC9441870

[16] Shimizu T Inoue E Ohkuma R Kobayashi S Tsunoda T Wada S. Soluble PD-L1 changes in advanced non-small cell lung cancer patients treated with PD-1 inhibitors: an individual patient data meta-analysis. Front Immunol. (2023) 14:1308381. doi: 10.3389/fimmu.2023.1308381, PMID: 38115995 PMC10728992

[17] Ancel J Dormoy V Raby BN Dalstein V Durlach A Dewolf M. Soluble biomarkers to predict clinical outcomes in non-small cell lung cancer patients treated with immune checkpoint inhibitors. Front Immunol. (2023) 14:1171649. doi: 10.3389/fimmu.2023.1171649., PMID: 37283751 PMC10239865

[18] Machiraju D Wieckenberg J Lang N Hülsmeyer I Roth J Schank TE. Soluble immune checkpoints and T-cell subsets in blood as biomarkers for resistance to immunotherapy in melanoma patients. Front Immunol. (2021) 12:663597. doi: 10.3389/fimmu.2021.663597, PMID: 34104542 PMC8158029

[19] Li YJ Msaouel P Campbell M Hwu P Diab A Kim ST. Successful management of pre-existing psoriatic arthritis through targeting the IL-23/IL-17 axis in cancer patients receiving immune checkpoint inhibitor therapy: a case series. RMD Open. (2024) 10:e004308. doi: 10.1136/rmdopen-2024-004308, PMID: 39214611 PMC11367333

[20] Wertheimer T Zwicky P Rindlisbacher L Sparano C Vermeer M de Melo BMS. IL-23 stabilizes an effector Treg cell program in the tumor microenvironment. Nat Immunol. (2024) 25:512–24. doi: 10.1038/s41590-024-01755-7, PMID: 38356059 PMC10907296

[21] Liu J Xing S Wang W Huang X Lin H Chen Y. Prognostic value of serum soluble interleukin-23 receptor and related cytokines in non-small cell lung cancer. Cancer Sci. (2020) 111:1044–53. doi: 10.1111/cas.14343, PMID: 32020720 PMC7156824

