# Supplementary material for: Cytokine profiling identifies circulating IL-2, IL23 and sPD-L1 as prognostic biomarkers for treatment outcomes in non-small cell lung cancer patients undergoing anti-PD1 therapy
Source: Front Oncol. 2025 Jul 8;15:1628379. doi: 10.3389/fonc.2025.1628379 (PMC12279494; doi:10.3389/fonc.2025.1628379)
Supplement: Supplementary file 1 [file DataSheet1.docx]

**Cytokine profiling identifies circulating IL-2, IL23 and sPD-L1 as prognostic biomarkers for treatment outcomes in Non-Small cell Lung Cancer patients undergoing anti-PD1 therapy**

Kriti Jain^1^, Anika Goel^1^, Deepa Mehra^1^, Deepak Kumar Rathore^2^, Akshay Binayke^2^, Shyam Aggarwal^3^, Surajit Ganguly^4^, Amit Awasthi^2^, Evanka Madan^1^*, Nirmal Kumar Ganguly^1*^

1. Department of Biotechnology and Research, Sir Ganga Ram Hospital, New Delhi, India
2. Immunology Lab, Translational Health Science and Technology Institute, Faridabad, India
3. Medical Oncology, Sir Ganga Hospital, New Delhi, India
4. Department of Molecular Medicine, Jamia Hamdard, New Delhi, India

**^*^Corresponding Authors-**

*Dr. Nirmal Kumar Ganguly Dr. Evanka Madan*

*Chairman Consultant & Assistant Professor*

*Department of Biotechnology and Research Department of Biotechnology and Research*

*Sir Ganga Ram Hospital Sir Ganga Ram Hospital*

[*ganguly1nk@gmail.com*](mailto:ganguly1nk@gmail.com) *evanka.madan@gmail.com*

**Supplementary Information**

**Table S1:** **Detailed Inclusion and exclusion criteria of patients enrolled in the study**

| **INCLUSION CRITERIA** | **EXCLUSION CRITERIA** |
| --- | --- |
| Patients with advanced, metastatic/ recurring NSCLC and are eligible for anti- PD-1 immune checkpoint therapy (nivolumab/pembrolizumab) were enrolled in the study. | Patients requiring concurrent anti-cancer therapy during the study period. Patients with communicable diseases (HIV, Hepatitis, etc). |
| Male and female patients in the age group of 18-80 years | Patients who are currently participating or had participated in clinical trials. |
| Patients willing to give informed consent | Patients on wysolove> 10 mg/day |
| Patients who are willing to comply with all study requirements | Patients with brain or subdural metastases are not eligible, unless they have completed local therapy and have discontinued the use of corticosteroids for this indication for at least 4 weeks before starting study treatment. |
| ECOG performance status≤ 2 | Uncontrolled intercurrent illness including, but not limited to ongoing or active infection, symptomatic congestive heart failure, unstable angina pectoris, recent myocardial infarction, cardiac arrhythmia, or psychiatric illness/social situations that would limit compliance with study requirements, or other comorbid condition that investigator believes may compromise participant’s condition |
| Women in reproductive age willing to follow adequate barrier contraceptive measures during the conduct of the study. | Patients who have received chemotherapy radiation or biological therapy within 2 weeks, or hormonal therapy within one week before study treatment start, or any investigational drug within 4 weeks before study treatment start. |
| Life expectancy > 4 months | Pregnant and lactating females |

**Table S1.** The table lists the detailed Inclusion and exclusion criteria of patients enrolled in this study.

**Table S2: Primer Sequences for Target Gene Amplification**

| **Target gene** |  | **Sequence** | **Tm (^o^C)** | **Amplicon size** |
| --- | --- | --- | --- | --- |
| IL-21 | Forward | 5’AGGTCAAGATCGCCACATGA 3’ | 59 | 172 bp |
| IL-21 | Reverse | 5’ TGCTGACTTTAGTTGGGCCT 3’ | 59 |  |
| PD-L1 | Forward | 5’ CACGGTTCCCAAGGACCTAT 3’ | 59 | 193 bp |
| PD-L1 | Reverse | 5’ GGCCCTCTGTCTGTAGCTAC 3’ | 59 |  |
| IL10 | Forward | 5’ TTAAGGGTTACCTGGGTTGC 3’ | 62.6 | 138 bp |
| IL10 | Reverse | 5’ TGAGGGTCTTCAGGTTCTCC 3’ | 63.2 |  |
| IL12 | Forward | 5’ ATGCCCCTGGAGAAATGGTG 3’ | 68.2 | 141 bp |
| IL12 | Reverse | 5’ GGCCAGCATCTCCAAACTCT 3’ | 65.6 |  |
| TNF-α | Forward | 5’ CCTCTCTCTAATCAGCCCTCTG 3’ | 63.2 | 220 bp |
| TNF-α | Reverse | 5’ GAGGACCTGGGAGTAGATGAG 3’ | 61.7 |  |
| IL32 | Forward | 5’ AGGCCCGAATGGTAATGCT 3’ | 65.5 | 84 bp |
| IL32 | Reverse | 5’ CCACAGTGTCCTCAG TGTCACA 3’ | 66.5 |  |
| GAPDH | Forward | 5’ GTCTCCTCTGACTTCAACAGCG 3’ | 54.5 | 131 bp |
| GAPDH | Reverse | 5’ ACCACCCTGTTGCTGTAGCCAA 3’ | 54.5 |  |
| IL-17 | Forward | 5’ AATCTCCACCGCAATGAGGA 3’ | 53 | 197 bp |
| IL-17 | Reverse | 5’ACCAGTATCTTCTCCAGCCG 3’ | 53 |  |
| PD-1 | Forward | 5’ CTCTGTGGACTATGGGGAGC 3’ | 57 | 199 bp |
| PD-1 | Reverse | 5’ AGAGCAGTGTCCATCCTCAG 3’ | 57 |  |
| IL-2 | Forward | 5’ AGGATCCCAAACTCACCAGGA 3’ | 58 | 168 bp |
| IL-2 | Reverse | 5’ TGCTGATTAAGTCCCTGGGT 3’ | 58 |  |

**Table S2:** The table lists the primer sequences used for amplifying specific target genes. Each gene has a designated forward (F) and reverse (R) primer, along with their respective melting temperatures (Tm) and amplicon size.

**Table S3:** **Association between cytokines levels and treatment response by univariate analysis**

| FACTOR | MEDIAN | ODD RATIO | P-VALUE |
| --- | --- | --- | --- |
| **AGE** | **65** | **1.144218 (1.048212-1.249018)** | **0.002587** |
| SEX | NA | 0.625 (0.22132-1.764981) | 0.374894 |
| HEIGHT | 168 | 1.037923(0.976085-1.103678) | 0.234975 |
| WEIGHT | 69 | 0.974616 (0.899249-1.056299) | 0.531215 |
| BMI | 25 | 0.85089 (0.69238-1.045688) | 0.124731 |
| DIET | NA | 1.777778(0.276459-11.432067) | 0.544553 |
| DIABETES MELLITUS | NA | 1.615385(0.597068-4.370472) | 0.344972 |
| HYPERTENSION | NA | 1.222222(0.443363-3.369306) | 0.698117 |
| ANY LUNG OR HEART DISEASES | NA | 1.184211(1.184211-3.205844) | 0.739324 |
| SMOKING | NA | 1.842105(0.655374-5.17773) | 0.246621 |
| ALCOHOL | NA | 1.272727(0.444954-3.640455) | 0.652887 |
| TOBACCO CHEWING | NA | 0.5625(0.087473-3.617178) | 0.544553 |
| **WEIGH LOSS** | **NA** | **3.174603(1.114912-9.039374)** | **0.030487** |
| PLEURAL EFFUSION | NA | 1.235294(0.454465-3.35769) | 0.678741 |
| PRE-TREATED RADIOTHERAPY | NA | 1.037037(0.305794-3.516901) | 0.953456 |
| PRE-TREATED CHEMOTHERAPY | NA | 0.738095(0.114817-4.744808) | 0.749061 |
| **HISTOLOGY** | **NA** | **2.955556(1.051512-8.30738)** | **0.039857** |
| STAGE | NA | 0.928571(0.339572-2.539212) | 0.885194 |
| METASTASIS | NA | 0.809524(0.297824-2.200391) | 0.678741 |
| **MICROSATELLITE INSTABILITY** | **NA** | **3.230769(1.156921-9.022108)** | **0.025211** |
| DESMOPLASIA COLLAGE III | NA | 3.57E+12(0-inf) | 0.99996 |
| **TP53P GENE MUTATION** | **NA** | **2.955556(1.051512-8.30738)** | **0.039857** |
| **TUMOR MICROENVIRONMENT BURDEN** | **8.775** | **1.232367(1.108194-1.370453)** | **0.000115** |
| **TNF-α** | **5.615161058** | **0.92918(0.866243-0.996689)** | **0.040107** |
| **PD-L1** | **0.513403** | **1.458845(1.052671-2.021742)** | **0.023313** |
| **IL-10** | **0.9996894169** | **0.436349(0.220681-0.862788)** | **0.017113** |
| IL-1β | 1.000162318 | 0.837578(0.652729-1.074775) | 0.163574 |
| IFN-ϒ | 0.6862981773 | 0.688172(0.325249-1.456055) | 0.328397 |
| IL-1α | 0.999620582 | 1.004545(0.452977-2.227734) | 0.991096 |
| IL-4 | 0.7833804353 | 0.91285(0.756402-1.101657) | 0.341795 |
| **IL-17** | **0.9998377747** | **17.62385(2.189363-141.867849)** | **0.00701** |
| **IL-2** | **1.000144817** | **18.26174(3.172607-105.115824)** | **0.001143** |
| GMCSF | 1.000296505 | 1.040435(0.878238-1.232587) | 0.646657 |
| **IL-13** | **0.9999981709** | **15.85767(1.34843-186.487872)** | **0.027972** |
| IL-15 | **4.394997553** | 1.01272(0.968247-1.059235) | 0.5812 |
| IL-21 | 1.000109511 | 0.656599(0.279912-1.540202) | 0.333508 |
| **IL-23** | **0.7759003153** | **0.78093(0.614897-0.991794)** | **0.042609** |
| IL-18 | 1.58785315 | 0.792579(0.424369-1.480274) | 0.465787 |

**Table S3:** This table provides the median values, odds ratios (HR) with 95% confidence intervals (CI), and p-values for each factor. Statistically significant p-values (< 0.05) are highlighted to denote factors potentially influencing Response Status.

**Table S4: Association of baseline cytokine levels and clinical variables with overall survival (OS) using univariate analysis**

| FACTOR | Median | Hazard Ratio | P Value |
| --- | --- | --- | --- |
| AGE | 65 | 0.996857(0.868984 - 1.143546) | 0.964151 |
| SEX | NA | 0.982675(0.292016 - 3.30684) | 0.97748 |
| HEIGHT | 168 | 1.020172 (0.895081 -1.162747) | 0.893568 |
| WEIGHT | 69 | 1.007823 (0.99098- 1.129695) | 0.93568 |
| BMI | 25 | 1.112859 (0.807099 - 1.534453) | 0.51413 |
| DIET | NA | 0.264332(0.02714 - 2.574524) | 0.251925 |
| **DIABETES MELLITUS** | **NA** | **8.578845(3.7844 -1.672298)** | **0.009986** |
| HYPERTENSION | NA | 1.118074(0.321007 - 3.894266) | 0.86085 |
| ANY LUNG OR HEART DISEASES | NA | 0.686727(0.786147 -0.214857) | 0.526135 |
| SMOKING | NA | 2.326875(0.648501- 8.349016) | 0.195124 |
| ALCOHOL | NA | 0.322775(0.069692 -1.494909 ) | 0.148213 |
| TABACCO CHEWING | NA | 0.550827 ( 1.494051 -0.068104 ) | 0.576074 |
| WEIGHT LOSS | NA | 1.626689( 0.416023 - 6.360515) | 0.48433 |
| PLEURAL EFFUSION | NA | 1.00156(1.353735 - 0.25908) | 0.998197 |
| PRE-TREATED RADIOTHERAPY | NA | 1.213673(1.353162 -0.380656 ) | 0.743414 |
| PRE-TREATED CHEMOTHERAPY | NA | 0.103418(0.508587 - 0.006431) | 0.109358 |
| HISTOLOGY | NA | 1.24764(1.380406 - 0.39145) | 0.708323 |
| METASTASIS | NA | 0.549133(0.170949 -1.763953) | 0.314062 |
| STAGE | NA | 0.925442(0.842538 -1.016503 ) | 0.105635 |
| TUMOR MICROENVIRONMENT BURDEN | 8.775 | 0.925442 (-0.171336 -0.016369 ) | 0.105635 |
| MICROSATELLITE INSTABILITY | NA | 0.499921 (0.760038 -0.116875 ) | 0.349797 |
| DESMOPLASIA COLLAGEN III | **NA** | **0.191239(0.039525 - 0.925294)** | **0.039735** |
| TP53P GENE MUTATION | NA | 0.319861(0.08327- 1.22867) | 0.096904 |
| **PD-L1** | **0.513403** | **0.7654567(0.45577-1.56789)** | **0.024437** |
| **TNF-α** | **5.615161058** | **1.218083 (1.000422 -1.4831)** | **0.049511** |
| IL-10 | 0.9996894169 | 1.336698(0.493558 - 3.620167) | 0.568075 |
| IL-1 β | 1.000162318 | 1.682801(0.010803 -262.143255) | 0.83987 |
| IFN-ϒ | 0.6862981773 | 1.5979(0.25546 -9.994853) | 0.616336 |
| IL-1α | 0.999620582 | 4.501389(0.703809 -28.789767) | 0.112068 |
| IL-4 | 0.7833804353 | 1.193862(0.963764 - 1.478896) | 0.104785 |
| IL-17 | 0.9998377747 | 1.083477(0.044898 - 26.146236) | 0.960632 |
| **IL-2** | **1.000144817** | **0.129847(0.0897497 - 0.1864796)** | **0.022994** |
| GMCSF | 1.000296505 | 1.85518(0.163942 - 20.99331) | 0.617623 |
| IL-13 | 0.9999981709 | 2.828918(0.070843 - 112.964826) | 0.580422 |
| IL-15 | 4.394997553 | 1.056368(0.989709 -1.127516 ) | 0.099165 |
| IL-21 | 1.000109511 | 3.049116(0.443462 -20.964862 ) | 0.257073 |
| **IL-23** | **0.7759003153** | **1.238954(1.191397 - 1.29042)** | **0.035139** |
| IL-18 | 1.58785315 | 0.940561(0.407267 - 2.172176) | 0.885902 |

**Table S4:** This table provides the median values, Hazard’s ratios (HR) with 95% confidence intervals (CI), and p-values for each factor. Statistically significant p-values (< 0.05) are highlighted to denote factors potentially influencing OS.

**Table S5:** **Association of baseline cytokine levels and clinical variables with progression-free survival (PFS) by univariate analysis**

| FACTOR | Median | Hazard ratio | *P-value* |
| --- | --- | --- | --- |
| AGE | 65 | 0.996857(0.868984 - 1.143546) | 0.964151 |
| SEX | NA | 0.982675 (0.292016- 3.30684) | 0.97748 |
| HEIGHT | 168 | 1.007823 (0.899098- 1.129695) | 0.893568 |
| WEIGHT | 69 | 1.020172 (0.895081- 1.162747) | 0.764761 |
| BMI | 25 | 1.112859(0.807099 - 1.534453) | 0.51413 |
| DIET | NA | 0.264332(0.02714- 2.574524 ) | 0.251925 |
| **DIABETES MELLITUS** | **NA** | **8.578845(3.7844- 1.672298)** | **0.009986** |
| HYPERTENSION | NA | 1.118074(0.321007 -3.894266 ) | 0.86085 |
| ANY LUNG OR HEART DISEASES | NA | 0.686727(0.786147 - 0.214857 ) | 0.526135 |
| SMOKING | NA | 2.326875(0.648501 - 8.349016 ) | 0.195124 |
| ALCOHOL | NA | 0.322775(0.069692 - 1.494909) | 0.148213 |
| TABACCO CHEWING | NA | 0.550827(0.068104 - 4.455106 ) | 0.576074 |
| WEIGHT LOSS | NA | 1.00156(0.416023 - 6.360515) | 0.48433 |
| PLEURAL EFFUSION | NA | 1.00156(0.25908 - 3.871862) | 0.998197 |
| PRE-TREATED RADIOTHERAPY | NA | 1.213673(1.353162 - 0.380656) | 0.743414 |
| PRE-TREATED CHEMOTHERAPY | NA | 0.103418(0.508587 - 0.006431) | 0.109358 |
| HISTOLOGY | NA | 1.24764(0.39145 -3.976516) | 0.708323 |
| STAGE | NA | 0.549133(0.170949 -1.763953 ) | 0.314062 |
| METASTASIS | NA | 0.549133(0.170949 - 1.763953) | 0.314062 |
| MICROSATELLITE INSTABILITY | NA | 0.499921(0.760038- 0.116875 ) | 0.349797 |
| DESMOPLASIA COLLAGEN III | **NA** | **0.191239(0.039525 - 0.925294)** | **0.039735** |
| **TP53P GENE MUTATION** | **NA** | **0.319861(0.08327- 1.22867 )** | **0.096904** |
| TUMOR MICROENVIRONMENT BURDEN | 8.775 | 0.777607(0.320169 - 1.888607) | 0.578506 |
| TNF-α | 5.615161058 | 1.094069(1.000422 - 1.4831) | 0.509273 |
| **PD-L1** | **0.513403** | **0.669887(0.570562 - 0.786503)** | **0.00509273** |
| IL-10 | 0.9996894169 | 1.336698 (0.493558 - 3.620167) | 0.568075 |
| IL-1β | 1.000162318 | 1.682801(0.01080 -262.143255) | 0.83987 |
| IFN-ϒ | 0.6862981773 | 1.5979(0.25546 - 9.994853) | 0.112068 |
| IL-1α | 0.999620582 | 4.501389(0.703809 -28.789767) | 0.112068 |
| IL-4 | 0.7833804353 | 1.193862(0.963764 - 1.478896) | 0.104785 |
| IL-17 | 0.9998377747 | 1.083477(0.044898 -26.146236) | 0.960632 |
| **IL-2** | **1.000144817** | **1.174337 (1.119104 -1.232295)** | **0.022994** |
| GMCSF | 1.000296505 | 1.85518(0.163942 - 20.99331) | 0.617623 |
| IL-13 | 0.9999981709 | 2.828918(0.07084 - 112.964826) | 0.58042 |
| **IL-15** | **4.394997553** | **1.056368(0.989709 -1.127516)** | **0.099165** |
| IL-21 | 1.000109511 | 3.049116(0.443462 -20.964862) | 0.257073 |
| **IL-23** | **0.7759003153** | **0.319092(0.107473 - 0.947396)** | **0.045139** |
| IL-18 | 1.58785315 | 0.940561(0.407267 - 2.172176) | 0.885902 |

**Table S5**: This table provides the median values, hazards ratios (HR) with 95% confidence intervals (CI), and p-values for each factor. Statistically significant p-values (< 0.05) are highlighted to identify factors potentially influencing PFS.
